# Supplementary material for: Interventions to control nosocomial transmission of SARS-CoV-2: a modelling study
Source: BMC Med. 2021 Aug 27;19:211. doi: 10.1186/s12916-021-02060-y (PMC8390112; doi:10.1186/s12916-021-02060-y)
Supplement: Supplementary file 1 — Additional file 1: Supplementary Material. (1) Data: Figure S1. Number of patients admitted to UMCU with a SARS-CoV-2 infection between 27 February and 2 August 2020. Figure S2. Length of stay data of UMCU and fitted distributions for non-COVID and COVID patients in the hospital. (2) Model: Figure S3. PCR test sensitivity over time since infection. (3) Calibration of parameters to data: Table S1. Model parameters. (4) Infection control interventions. (5) Implementation of the model: Figure S4. Overview of processes in the agent-based model. Figure S5. Flowchart for patient arrival and patient discharge in the agent-based model. Figure S6. Flowchart for HCW community transmission in the agent-based model. Figure S7. Flowchart for HCW ward change in the agent-based model. Figure S8. Flowchart for HCWs meeting in common room in the agent-based model. Figure S9. Flowchart for transition of disease states in agent-based model. Figure S10. Flowchart for HCWs visiting patients in the agent-based model. Figure S11. Flowchart for contact tracing in the agent-based model. Figure S12. Flowchart for HCW screening in the agent-based model. [file 12916_2021_2060_MOESM1_ESM.docx]

**Additional File 1: Supplementary methods for the article *Interventions to control nosocomial transmission of SARS-CoV-2: a modelling study***

Thi Mui Pham^1,*,#^, Hannan Tahir^1,*^, Janneke H.H.M. van de Wijgert^1,2^, Bastiaan R. Van der Roest^1^, Pauline Ellerbroek^3^, Marc J.M. Bonten^1,5^, Martin C.J. Bootsma^1,4^, Mirjam E. Kretzschmar^1^

^1^ Julius Center for Health Sciences and Primary Care, University Medical Center Utrecht, Utrecht University, Utrecht, The Netherlands

^2^ Institute of Infection, Veterinary, and Ecological Sciences, University of Liverpool, Liverpool, UK

^3^ Department of Internal Medicine, University Medical Center Utrecht, Utrecht University, Utrecht, The Netherlands

^4^ Department of Mathematics, Faculty of Science, Utrecht University, Utrecht, The Netherlands

^5^ Department of Medical Microbiology, University Medical Center Utrecht, Utrecht University, Utrecht, The Netherlands

**^*^ These authors contributed equally to this work.**

**^#^ Corresponding author:**

Thi Mui Pham

Address: Julius Center for Health Sciences and Primary Care

University Medical Center Utrecht

P.O. Box 85500 Utrecht

The Netherlands

E-mail: [t.m.pham-2@umcutrecht.nl](mailto:t.m.pham-2@umcutrecht.nl)

Telephone: +31648938724

**Supplementary methods**

We simulated nosocomial COVID-19 epidemics using an agent-based model coded in Python. The code is available from: <https://github.com/htahir2/covid_intra-hospital_model.git>. First, we fitted the model to real-life data from the University Medical Center Utrecht (UMCU) during the period February-August 2020. Next, we evaluated the impact of various intervention strategies aimed at healthcare workers (HCWs) on the nosocomial spread of a more transmissible SARS-CoV-2 variant (e.g., B.1.1.7) in a hospital.

We first outline the data used to inform and parametrize our model. We continue by explaining the details of our agent-based model, the transmission model, and the underlying assumptions. We further describe the intervention strategies implemented in our model, the considered outcome measures, and the results of our sensitivity analyses. Lastly, we elaborate on the algorithm of our model.

1. Data

We used data from the University Medical Center Utrecht (UMCU), The Netherlands, and data provided by the National Institute for Public Health and the Environment (RIVM), The Netherlands, during the first wave of the SARS-CoV2 epidemic to inform and parametrize our model.

*Hospitalization data*

We used unlinked anonymized hospitalization data of patients in COVID wards at the UMCU between 27 February and 24 August 2020. The data set comprises 167 admissions of which 82 patients were admitted to an intensive care unit, and 85 patients were admitted to regular wards. Based on information of the infection control department of the UMCU, we assumed that 95% of those admissions were COVID-19 admissions, leaving 5% of the patients admitted for non-COVID reasons but later diagnosed with a SARS-CoV-2 infection in the hospital. The number of admissions per day are shown in Figure S1. The data further comprises discharge dates of the respective patients. We used the resulting number of beds occupied by COVID-19 patients to fit the reproduction numbers in our model. The data is available from: <https://github.com/htahir2/covid_intra-hospital_model/blob/main/data/covid_patient_admissions_los_UMCU.csv>

*Length of stay distributions*

We calculated the respective length of stay (LoS) from admission and discharge times for each of the COVID-19 patients at UMCU. Data on patient admissions to the UMCU prior to the COVID-19 pandemic (2014-2017) were used to estimate the length of stay distributions for non-COVID admissions to the hospital. We fitted probability distributions to the length of stay data for admissions to regular wards and ICUs, separately for COVID or for non-COVID related admissions. We considered exponential, log-normal, gamma, and Weibull distribution and chose the best fit by visual inspection of the empirical vs theoretical densities, the Q-Q plot, and the P-P plot. The length of stay data and fitted distributions are shown in Figure S2. The respective parameters can be found in Table S1.

*Importation from community*

We assume that 40 patients are admitted to the hospital for reasons unrelated to COVID-19 per day in the time period 27 February to 24 August 2020 (Table S1). We based this number on UMCU admission data in the time period 2014-2017 and the assumption that admissions decreased by 50% during the first wave of the COVID-19 epidemic. Those admitted patients might be infected with SARS-CoV-2 due to transmissions in the community. We further assume that HCWs go home after each daily shift and therefore may acquire infection in the community as well. They may be in their pre-symptomatic phase or asymptomatically infected with SARS-CoV-2 when they arrive at work in the hospital. These patients and HCWs do not experience any symptoms (yet), and therefore do not know that they are infected. We approximate the probability of being infected in the community for non-COVID patient admissions and HCWs arriving at work as follows:

We used data on the number of infectious people in the Netherlands estimated by the National Institute for Public Health and the Environment (RIVM) from 17 February till 24 August 2020 [17]. They used data from serological surveys in the Netherlands and related these to numbers of hospitalized cases (stratified by age group), leading to the number of “actual” infections per hospitalized case. They thereby included all infections that led to an immunological response, not only those that were detected in real time by PCR testing. Note that this method might be less reliable when the number of hospitalizations is low. This estimated number of infectious individuals includes hospitalized COVID-19 patients as well as individuals that are isolated at home (e.g., due to detection in the community via testing or contact tracing). To roughly account for this, we subtracted the total number of reported cases in the province of Utrecht from the estimated number of infectious individuals (RIVM estimate described before). We hereby assume that all individuals in Utrecht are eligible for admission at the UMCU. We additionally used publicly available age-specific hospitalization rates of the Netherlands of 2012 and age-specific COVID-19 incidence rates in the Netherlands to scale the daily probability of being infected in the community for non-COVID patient admissions and HCWs arriving at work [18,19,32]. For HCWs, we only used age-specific COVID-19 incidence rates for age-groups between 20 and 65 years. Since age-specific prevalence values are not available to date, the previous calculation is based on the assumption that the distribution of age groups is roughly the same for incidence and prevalence. Furthermore, we assumed a catchment population size of 100,000 people for the hospital.

1. Model

*Environment*

We modelled a typical (Dutch) hospital comprising 28 wards which are divided as follows

- COVID ICU (4) with 17 beds each
- Normal (non-COVID) ICU (1) with 12 beds
- COVID ward (4) with 3 x 23 beds and one with 22 beds
- Normal (non-COVID) ward (19) with 2 x 20, 4 x 19, 13 x 18 beds

The total number of beds in the hospital is 521. The numbers are approximated in accordance to the number of beds and ward distribution of the UMCU (for patients who stayed at least one day at UMCU).

*Agent-types*

There are three different agents involved in the transmission process within the hospital of our model: Patients (non-COVID and COVID) and health-care workers (HCWs), separated into nurses, and doctors. Visitors or ancillary workers are excluded from the model.

Patients are assumed to occupy a hospital bed in a single room. This assumption is suitable for a setting where the transmission is mainly driven by HCWs as vectors and with no direct patient-to-patient transmission. HCWs have a number of duty shifts per day. We assume that they meet patients in a number of rounds per shift (see Table S1) and that HCWs meet other HCWs in the common staff room of each ward.

The ratio between HCWs and patients and the time HCWs spend with a patient are ward specific and assumed. The number of HCW duty shifts per day, and the number of rounds per shift are independent from the ward and assumed. The respective parameters can be found in Table S1.

*Disease progression*

The disease progression of an infection with SARS-CoV-2 was modelled using a Susceptible-Exposed-Infectious-Recovered (SEIR) model and is shown in Figure 1c of the main text. Individuals who have not been infected with SARS-CoV-2 are susceptible (S), and may transition to being exposed (E) upon contact with an infected individual. A proportion ($1-P_{A}$) of infected individuals develop symptoms. We based the incubation period (time between infection and appearance of symptoms) on a Gamma distribution with mean 5.5 days as described by Lauer et al [15]. Symptomatically infected individuals may develop moderate symptoms (I_M_) or severe symptoms (I_S_). All infected individuals will eventually recover and become immune (I_R_). Asymptomatically infected (I_A_) are assumed to recover after 14 days. We assume that moderately and severely infected patients recover after 14 and 35 days, respectively [16]. We assumed that symptomatically infected HCWs are perfectly isolated at home for seven days immediately upon developing symptoms, after which they return recovered to work. Based on a meta-analysis by Buitraga-Garcia et al, we assumed the asymptomatic proportion of COVID-19 infections among patients to be 20% and the proportion of asymptomatic infections among HCWs to be 31% (see also Table S1) [20]. We used their overall estimate of the proportion of asymptomatic infected individuals for the patient population in our model, and their estimates obtained from studies with screened individuals for the HCW population in our model.

*Hospital admissions*

Patients can be hospitalized either for non-COVID reasons to normal wards and ICUs, or with moderate or severe COVID-19 symptoms to COVID wards or COVID-ICUs. The length of stay of a patient differs according to these four categories. Probability distributions were fitted to length of stay data of patients admitted to the UMCU. The data and fitted distributions are shown in Figure S1-S2. The respective parameters can be found in Table S1. Patients admitted to normal wards and ICUs who are later detected with a SARS-CoV-2 infection are immediately transferred to COVID wards and ICUs upon diagnosis. If they develop severe symptoms, their length of stay is prolonged according to the length of stay distribution of admitted severe COVID-19 patients.

*Accuracy of the diagnostic test*

In our model, patients and HCWs are assumed to be tested using reverse transcriptase polymerase chain reaction (RT-PCR) either when being screened or after being identified as a contact of a symptomatic infected individual in contact tracing. These diagnostic tests can be inaccurate either because of a false positive or a false negative result. The latter are considered to be more consequential with a potential high impact on onward transmission due to undetected cases. It has been documented that the sensitivity of the PCR test varies with time from exposure and symptom onset [33]. We assumed a time-varying imperfect sensitivity of the diagnostic test (Figure S3) based on the results reported in Grassly et al [14]. These authors used published data from three meta-analyses of the test sensitivity over time since symptom onset. They assume the pre-symptomatic sensitivity to be proportional to the infectiousness curve such that the estimate on day 5 matched the empirical data from the day of symptom onset. We performed a sensitivity analysis assuming 1) a 15% lower sensitivity, and 2) a test sensitivity that stays at its peak value after reaching the maximum (see Figure S3). Finally, we assessed the impact of perfect sensitivity of 100% which stays constant over time on the results of our model. Throughout the simulations, we assume test sensitivity to be the same for symptomatic and asymptomatic infections, and we assume a specificity of 100%.

*Infectiousness*

We use a time-varying infectiousness profile following Grassly et al and shown in Figure 1C of the main text [14]. Infectiousness is assumed to vary over time since infection and to follow a Weibull distribution, with a mean of 6 days [14]. The average duration of the infectious period is therefore assumed to be 6 days. This approximation is consistent with published estimates of the serial interval for SARS-CoV-2 [34–36]. We denote infectiousness over time since infection $\tau$ by $\beta\left( \tau\right)$. It is the mean rate at which an individual infects others at time $\tau$ after its time of infection. We use the infectiousness profile for calculating the probability of transmission from an infectious to a susceptible individual (see below). The reproduction number $R$ (average number of secondary cases caused by an infected individual) is given by integrating ­$\beta(\tau)$ over time since infection $R=\int_{0}^{\infty} \beta\left( \tau\right)d\tau$. The generation time distribution $\omega(\tau)$ is given by unit normalisation such that $\omega\left( \tau\right)=\beta\left( \tau\right)/R$. Assuming the mean generation time to be equivalent with the observed mean serial interval, we calculate the infectiousness profile by ­$\beta\left( \tau\right)=\omega\left( \tau\right)R.$ We assumed the infectiousness over time since infection to differ between asymptomatic and symptomatic infected individuals, defined by$\beta_{A}(\tau$) and $\beta_{S}(\tau)$, respectively. Then $\beta(\tau)$ can be decomposed into

$$\beta\left( \tau\right)=P_{A}\beta_{A}\left( \tau\right)+\left( 1-P_{A} \right)\beta_{S}(\tau)$$

where $P_{A}$ represents the proportion of asymptomatic infections. Asymptomatic individuals are assumed to have an infectiousness proportional to that of symptomatic individuals, i.e., $\beta_{A}=x_{A}\cdot\beta_{S}, x_{A}\leq1$. Integrating over each of the two terms leads to the respective contribution to the overall reproduction number:

$$R=R_{A}+R_{S}$$

First, we chose the basic reproduction numbers$R_{S}$ and $R_{A}$ (values are given in Table S1) such that the numbers of occupied beds by COVID-19 patients predicted by our model were in good agreement with real-life UMCU data on the number of COVID-19 patients at UMCU during the first epidemic wave (Figure 1 and Figure 2A in the main text). These reproduction numbers incorporated the effects of typical (but not COVID-specific) infection prevention measures in the hospital. We will refer to the model parameterized with these reproduction numbers as the *wild-type scenario*. This scenario also assumed that HCWs use 90% effective PPE in COVID wards and isolate at home immediately upon symptom onset for seven days, after which they return recovered to work. Next, we introduced a more transmissible SARS-CoV-2 variant into the hospital, keeping all other parameters – including PPE use in COVID wards and self-isolation after symptom-onset – the same. Based on recent results for B.1.1.7, we assumed a 56% increase in transmissibility [25]. We will refer to the model parametrized with these higher reproduction numbers as our *baseline scenario*. Various intervention scenarios were compared to this baseline scenario.

*Transmission*

Transmission events can occur between susceptible patients and HCWs, or between (asymptomatic or pre-symptomatic) HCWs. Thus, we assumed that there is no direct transmission between patients. Only HCWs in their pre-symptomatic stage, or HCWs who are asymptomatically infected, contribute to transmission, since we assume that HCWs are perfectly isolated at home for seven days immediately upon developing symptoms. Upon a contact between two individuals, a transmission may take place between an infected and a susceptible individual. The probability of transmission is dependent on the current infectiousness of the infected individual. If $\beta(\tau)$ is the infectiousness of the infected individual at time $\tau$ since infection, the average probability of transmission per contact with a susceptible person is given by

$$A\left( \tau\right)=\frac{\beta(\tau)}{c}$$

where $c$ is the average contact rate of the individual which can be determined by computing the largest eigenvalue of the respective contact matrix

$$M=\left[ \left. \begin{matrix} c_{n,n} & c_{n,p} & c_{n,d} \\ c_{p,n} & 0 & c_{p,d} \\ c_{d,n} & c_{d,p} & c_{d,d} \end{matrix} \right] \right.$$

where $c_{i,j}$ is the contact rate of an individual of type $i$ with an individual of type $j$ (see Table S1). Let $N_{n}, N_{hc},$ and $N_{p}$ be the average number of nurses, doctors, and patients in the hospital population, respectively. Since the total number of contacts of individuals of type $i$ has to be the same as the total number of contacts of an individual of type $j$, $c_{n,p}N_{n}=c_{p,n}N_{p}$, $c_{d,p}N_{p}=c_{p,d}N_{d}$, and $c_{d,n}N_{n}=c_{n,d}N_{d}$ the contact matrix is given by

$$M=\left[ \left. \begin{matrix} c_{n,n} & c_{n,p} & c_{n,d} \\ c_{n,p}\frac{N_{p}}{N_{n}} & 0 & c_{p,d} \\ c_{n,d}\frac{N_{d}}{N_{n}} & c_{p,d}\frac{N_{d}}{N_{p}} & c_{d,d} \end{matrix} \right] \right.$$

The values of the contact rates in the matrix are based on HCW to patient ratios and the number of rounds per shift of HCWs and were estimated once from our simulations assuming 100% patient occupancy in the hospital. These estimated parameters were later used for all the simulation scenarios. The respective values can be found in Additional File 1: Table S1. We compared these values to a prospective contact survey of nurses in five hospitals in the German federal state of Bavaria conducted by Bernard et al [37]. The authors reported a median work-related contact rate of $c_{n}=34$ of nurses during 24 hours. Most work-related contacts were with patients (51%) or other staff member/other persons (49% = 40% + 9%). Thus, nurses meet approximately 17.3 patients and 16.7 other staff members per 24 hours. The contact rate of nurses with patients per duty shift from our simulations ($c_{n,d}$=19·07) is similar to the reported value by Bernard et al. The contact rates between hospital staff are lower in our simulation than reported in the contact survey (see values in Additional File 1: Table S1) and based on our assumption that contacts between hospital staff decreased during the first wave of the COVID-19 pandemic in the Netherlands.

*Time of infection*

For individuals infected in the community, the time of infection is unknown. For asymptomatic individuals, we assume an infectious period of 14 days and draw the infection time uniformly from 0 to 14 days prior to admission. For individuals that will develop symptoms, we draw an incubation period $t_{inc}$ from the respective distribution (see Table S1) and then draw the infection time uniformly from 0 to $t_{inc}$ prior to admission. Note that this approach neglects the fact that in an early stage of an outbreak when the epidemic grows at an exponential rate, it is likely that there are many more recently infected individuals.

1. Calibration of parameters to data

In such a multi-dimensional parameter space, it is possible that there are multiple sets of parameters that would produce the fit. The parameter set we presented in this work is likely not the only one that could produce the presented fit (Figure 2A in the main text). For many parameters we have knowledge from empirical observations about the likely values for those parameters (e.g., incubation period, generation time, …). This already considerably limits the parameter region of the model. We then tried to identify the parameters that would most likely change the fit to the data. For example, when we extended the length of stay of hospital-acquired infected patients with mild symptoms, the simulation output did not fit to the data anymore (“width” of the curve did not fit). This confirmed information we received from UMC Utrecht that in the Netherlands, the LoS of patients who do not require intensive care would not be extended (but patients would be sent home for isolation). Similarly, we noticed that when we changed the reproduction number that this had a big impact on the “height” of the curve. Thus, we calibrated the basic reproduction numbers such that the height of the simulation curve matched the data (and the width matched already with the remaining parameters given in Table 1 in the main text and Additional File 1: Table S1). Fine-tuning could yield a better fit but this is out of scope of this work.

The following parameters were involved in the calibration of the parameters to the observed data on occupied beds of COVID-19 patients at UMC Utrecht:

- Reproduction numbers for asymptomatically and symptomatically infected individuals
- Length of stay adjustment for patients who acquired a SARS-CoV-2 infection in the hospital
- Isolation period of HCWs

1. Infection control interventions

We used the model to evaluate the effect of several interventions aimed at HCWs on the hospital epidemic using data from the first wave of the epidemic in the Netherlands but assuming the introduction of a SARS-CoV-2 variant with higher transmissibility in the hospital. As such, our model results show the impact of the interventions on the nosocomial spread of a new variant.

Throughout the simulations, we assume that HCWs use 90% effective PPE in COVID wards and isolate at home immediately upon symptom onset for seven days, after which they return recovered to work. Furthermore, we assume that there is no delay between testing and receiving the COVID-19 test result. This assumption is in particular reasonable for hospital staff tested in the hospital, as they receive their test result within hours (UMCU) and have to self-isolate until they receive the result. Thus, it can be assumed that they do not contribute to the transmission of the virus while waiting for the test result.

*Baseline scenario*

We assumed that HCWs used personal protective equipment (PPE) while working in COVID wards. PPE reduces the transfer of droplets or other body fluids onto HCWs’ skin and clothing or directly onto the mucous membranes of the eye or nasopharynx. We define PPE efficacy as the percentage reduction of droplet transfer. Furthermore, we define the effectiveness of PPE as the reduction of infectiousness by a factor upon each contact between an infected and susceptible individual. This reduction factor includes PPE efficacy as well as adherence to adequate PPE use. In our baseline scenario, we assumed that all PPE use was 90% effective. We assumed HCWs do not use PPE when meeting each other in the common room and that per day 95% of the HCWs work in the same ward as during their previous shift.

*Intervention: PPE in all hospital wards*

In this intervention scenario, we assumed that all HCWs wore 90% effective PPE in all (non-COVID and COVID) wards. Note that no PPE is worn when HCWs meet each other. We performed sensitivity analyses assuming PPE effectiveness of 50% and 70%.

*Intervention: HCW cohorting (no HCW ward change)*

In this intervention scenario, we restricted HCWs to work only in specific wards and did not allow any ward change.

*Intervention: Regular HCW screening*

All HCWs were tested for SARS-CoV-2 either with a) a test with perfect sensitivity every three days, or a test with time-varying sensitivity, b) every three days, or c) every seven days. If tested positive, HCWs were assumed to immediately self-isolate at home for seven days.

*Intervention: HCW contact tracing*

If a HCW developed symptomatic SARS-CoV-2 infection, all contacts in the hospital during a time window of either two or seven days before symptom onset were traced and tested. We will refer to these scenarios as *2-day Contact tracing* and *7-day contact tracing*. For *2-day contact tracing*, contacts were always tested assuming a time-varying test sensitivity. For *7-day contact tracing*, we distinguished between perfect and time-varying sensitivity sub-scenarios. In the perfect sensitivity sub-scenario, contacts were instantaneously tested on the day of symptom onset of the index (the HCW). In the time-varying test sensitivity sub-scenario, the test was performed on the day of symptom onset if the contact with the index was more than five days ago. Otherwise, it was performed on day five after the contact. Exposed HCWs awaiting tests were assumed to wear PPE during contact with any patient and with other HCWs. In case of a positive test, patients were moved to a COVID ward while infected HCWs were sent home for self-isolation for seven days and replaced by susceptible HCW. We did not model any absences of HCWs with disease symptoms caused by other respiratory pathogens.

*Outcome measures*

We calculated the effective reproduction number $R_{E}$ (average number of secondary cases caused by an infected individual) to evaluate an intervention’s effectiveness in suppressing outbreak expansion in the hospital. We approximated $R_{E}$ for an average individual (patients and HCWs combined) in the hospital (overall $R_{E}$) from our simulations by calculating the average number of secondary cases by an infected individual in our model. We further stratified this number by patients, HCWs, and symptom status. The reproduction numbers of patients were calculated for those who will eventually develop symptoms $(R_{S}^{pat})$ and those who will remain without symptoms $(R_{A}^{pat})$. Since HCWs were assumed to immediately self-isolate upon symptom onset, we calculated $R$ during pre-symptomatic ($R_{S}^{hcw}$) and asymptomatic states ${(R}_{A}^{hcw})$ only. In order to evaluate the maximum demand on the hospital capacity, we considered the total number of hospital-acquired infections among patients and HCWs over time. In addition, we computed the proportion of absent HCWs due to self-isolation (because of symptom onset or detection via screening or contact tracing) over time. We assessed the efficiency of screening and contact tracing interventions with respect to their positivity rates (proportion of detected infected individuals among tested individuals). We did not include individuals that developed symptoms prior to being tested in the positivity rate calculations since those were already detected and isolated in our model. We determined the proportion of transmissions attributed to different transmission routes (HCW-to-HCW, HCW-to-patient, patient-to-HCW). For every scenario, we calculated the mean and 95% percentiles over 100 simulation runs (95% uncertainty interval). We calculated positivity rates over time merging data from all simulation runs and computed 95% Bayesian beta-binomial credibility intervals.

1. Implementation of the model

The model was built using *Python* (version 3.6) using the library *Mesa* which is an open source agent-based modelling framework [38]. The code is available from: <https://github.com/htahir2/covid_intra-hospital_model.git>

An overview of all processes in the implemented model is shown in Figure S4. The individual processes are described below and in flowcharts in Figure S5-S12.

*Initialization*

The model is initialized with non-COVID patients admitted to normal ICU and normal wards. We assume 50% of the rooms (beds) in the normal ICU and normal wards are free at the moment of model initialization. There are three duty shifts in a day and in these duty shifts, HCWs (nurses and doctors) are assigned to all wards in the hospitals. We assume that the number of HCWs in the hospital remains constant throughout the simulation period. Every patient agent has its own unique characteristics such as ID, ward and room number, LoS, and disease state. Depending on the ward (normal ICU or normal ward), every patient is assigned a LoS from the given distributions at the time of admission (Table S1). HCWs also have unique characteristics such as ID, ward to which HCW is assigned, duty shift in which HCW is working, HCW wearing PPE or not, time being absent from work due to quarantine, and disease state. A patient or a HCW can only be in one of the following disease states at a particular moment: susceptible, exposed, moderate symptomatic, severe symptomatic, asymptomatic, or recovered. However, at the model initialization, all patients and HCWs are in susceptible state.

*Study period*

The simulation is run for 239 days in total, with an initial period of 59 days to get a stable non-COVID patient population in the hospital. The first symptomatic COVID-19 patient is admitted to the hospital on day 60.

*Scheduling*

A time step in our agent-based model represents 10 minutes. The following processes occur during the run time:

*New patient arrival*

Given an average daily patient arrival rate for the UMCU (Table S1), patients arrive at the hospital following a Poisson process and are randomly admitted to normal ICU and normal wards. The majority of these new daily patients are in a susceptible state but as mentioned earlier in the section “*Importation from community*”, we use a community-prevalence-dependent, age-specific importation rate of exposed and asymptomatic patients into the hospital. Therefore, some patients from the daily new patient arrivals come in an exposed or asymptomatic state. Since the disease status of such patients is not known at the time of admission, they are admitted to normal ICU or normal wards. Depending on the patient ward (normal ICU or normal ward), the LOS is drawn from the appropriate distributions (Table S1).

The first symptomatic COVID-19 patient is admitted to the hospital on day 60 (based on UMCU COVID-19 admission data). Known symptomatic patients are admitted to either COVID wards or COVID ICUs depending on the severity of their symptoms. Moderate symptomatic patients are admitted to COVID wards, whereas severe symptomatic patients are admitted to COVID ICUs. For symptomatic COVID patients admitted to the hospital, LOS of the individual patient is sampled directly from the UMCU data.

*Patient discharge*

The remaining LOS of every patient is decremented at every time step. When the LOS of a patient reaches zero, the patient is discharged from the hospital. We do not model patient deaths.

*HCWs visiting patients*

At each time step, HCWs (nurses and doctors) from every ward visit individual patients. This is the moment where a contact between HCW and patient takes places. Single HCWs visit one single patient in one time step. When a HCW and a patient meet in a room and if one of them (patient or HCW) is infected with SARS-CoV-2, a transmission event can take place. As explained earlier in the section “*Transmission*”, a Bernoulli trial using the average probability of transmission per contact with a susceptible person is carried out. If a trial is successful, a susceptible individual acquires infection. The patient may be in an exposed state and develop symptoms after an incubation period or be asymptomatically infected. All exposed individuals in the model follow symptomatic route whereas asymptomatic individuals follow asymptomatic route. To decide on this for a patient, a random number is drawn and if it is less than the specified proportion of asymptomatic patients ($P_{A}^{p}$), the patient state is changed to asymptomatic, otherwise exposed. Similarly, for a HCW if the random number is smaller than the specified proportion of asymptomatic HCWs ($P_{A}^{h}$), the HCW’s state is changed to asymptomatic, otherwise the state of the susceptible HCW is changed to exposed. Infectiousness from symptomatic or asymptomatic individuals over time is different as explained earlier in the section “Infectiousness”. For exposed patients and HCWs, an incubation period $t_{inc}$ is drawn from the Gamma distribution ($s(\tau)$) with a mean of 5.5 days.

*Exposed to infection*

For every individual in an exposed state, the incubation period is then decremented by 1 at every time step. When the incubation time of an exposed individual (patient or HCW) reaches zero, the individual is either moved to moderate or severe symptomatic state depending on the proportion of individuals developing severe symptoms ($P_{s}$).

For patients who develop severe symptoms, a sample is drawn from the LoS of severely infected patients (based on UMCU data) and the LoS of the respected patient is extended accordingly. If the drawn LoS is shorter than the original one, the original LoS remains unchanged. The LoS of moderately infected patients is not extended. This is based on the assumption that those patients do not require intensive care and they are sent home for isolation. Severely infected patients are then moved to one of the COVID-ICUs, whereas moderately infected patients are moved to any of the COVID wards.

A HCW who develops symptoms is sent home for an isolation period of seven days, and a susceptible HCW is added in the same ward and shift as a replacement.

*Infection to recovery*

Moderately or severely infected patients are assumed to recover either at the time of discharge or after a maximum period of 14 days or 35 days, respectively. Asymptomatically infected patients recover at the time of discharge or after maximum period of 14 days.

Moderately or severely infected HCWs return to work as recovered after the end of the isolation period of seven days. When a recovered HCW returns to work to a specific ward, a HCW is removed from that ward as we assume a constant HCW population. To do that, we first look in the list of susceptible HCWs in the same ward and duty shift. If that list is not empty, a susceptible HCW is randomly chosen and removed from the hospital HCWs population. If there is no susceptible HCWs in that ward and duty shift, we look further into the list of exposed and asymptomatic HCWs in the same ward and duty shift. If that list is not empty, then a randomly chosen exposed or asymptomatic HCW is removed from the hospital population. If this is also not successful, we randomly choose a recovered HCW from the same ward and duty shift and remove him from the hospital population. These steps are required to maintain a constant HCW population.

*HCWs meeting in the common areas*

Every hour, two HCWs meet in the common areas of every ward. For this, we randomly pick two HCWs from the list of HCWs working in a ward in a shift. If one of the randomly chosen HCW is exposed or asymptomatic and the other HCW is susceptible, a transmission event can take place. As explained earlier in the section “*HCWs visiting patients*”, a Bernoulli trial using the average probability of transmission per contact with a susceptible person is carried out. If the trial is successful, the susceptible HCW acquires infection and can either enter into an exposed state or an asymptomatic state. Next, a random number is drawn, and if smaller than the specified proportion of asymptomatic HCWs ($P_{A}^{h}$), the HCW state is changed to asymptomatic, otherwise the state of the HCW is changed to exposed. For an exposed HCW, an incubation period $t_{inc}$ is drawn from the Gamma distribution ($s(\tau)$) with a mean of 5.5 days.

*HCWs ward swap*

Before the start of each day, a certain proportion of HCWs ($W_{h}$) are randomly selected and their wards are changed (Table S1). In order to do that, we first loop over the list of active HCWs (the ones that are not isolated at home). Since we change wards of two HCW at the same time (ward swapping), we draw a random number for every HCW. If the random number is less than $W_{h}/2$, we select that specific HCW (HCW-A) to be moved to another ward. The next step is to find another HCW (HCW-B) in a ward different from the ward of HCW-A. For this, we again make a list of all the active nurses (if HCW-A was a nurse) or active doctors (if HCW-A was a doctor), and then randomly pick a HCW (HCW-B). Once we have selected both HCW-A and HCW-B, we can now swap the wards, duty shifts and PPE of the both HCWs. We repeat the above process for all active HCWs.

**Table S1. Model parameters**

| Name | Symbol | Description | Distribution/Value* | Source |
| --- | --- | --- | --- | --- |
| Incubation period | $s(\tau)$ | Time between infection and symptom onset | Gamma distribution  shape=5.807  scale = 0.948  mean = 5.510  SD = 2.284 | Lauer et al [15] |
| Generation time | $\omega(\tau)$ | Time between becoming infected and subsequent onward transmission events | Weibull distribution  shape = 2.826  scale = 6.839  mean = 6 days | Grassly et al [14] |
| Proportion of asymptomatic infections among infected patients | $P_{A}^{p}$ | Proportion of infected patients that will experience no symptoms | 20% | Buitraga-Garcia et al [20] |
| Proportion of asymptomatic infections among infected HCWs | $P_{A}^{h}$ | Proportion of infected HCWs that will experience no symptoms | 31% | Buitraga-Garcia et al [20] |
| Proportion of severe symptomatic individuals | $P_{s}$ | Proportion of exposed individuals that will develop severe symptoms | 20% | Wu et al [21] |
| Reproduction number of asymptomatic infectees for wild-type variant | $R_{A}^{W}$ | Mean number of infections caused by an individual asymptomatically infected with the wild-type SARS-CoV-2 variant | 0.5 | Calibrated to UMCU data |
| Reproduction number of symptomatic infectees for wild-type variant | $R_{S}^{W}$ | Mean number of infections caused by an individual symptomatically infected with the wild-type SARS-CoV-2 variant | 1.25 | Calibrated to UMCU data |
| Reproduction number of asymptomatic infectees for new virus variant | $R_{A}$ | Mean number of infections caused by an individual asymptomatically infected with the SARS-CoV-2 variant | 0.8 (1.95) | Based on $R_{A}^{W}$ with 56% higher transmissibility, varied in sensitivity analysis |
| Reproduction number of symptomatic infectees for new virus variant | $R_{S}$ | Mean number of infections caused by an individual symptomatically infected with the SARS-CoV-2 variant | 1.95 | Based on $R_{A}^{W}$ with 56% higher transmissibility |
| Peak sensitivity of RT-PCR test for SARS-CoV-2 |  | Maximum sensitivity of the RT-PCR diagnostic test for SARS-CoV-2 | 93.1% (79%) | Grassly et al [14], varied in sensitivity analyses |
| Proportion of HCWs that work in the same ward as during their previous shift | $W_{h}$ | Proportion of HCWs that change wards they were working in their previous shift | 95% (baseline)  100% (intervention) | Assumed |
| PPE effectiveness |  | Reduction in infectiousness upon contact between an infected and susceptible individual (includes PPE efficacy and adherence) | 90% (50%, 70%) | Assumed |
| Isolation period for HCWs |  | Amount of time HCWs have to isolate after symptom onset or after being detected by screening or contact tracing | 7 days | Assumed |
| Recovery time for asymptomatic infection | $\gamma_{A}$ | Mean duration of an asymptomatic infection | 14 days  Sensitivity analysis: Uniform(9,19) | Assumed |
| Recovery time for symptomatic (moderate, severe) infection | $\gamma_{S}$ | Mean duration of a symptomatic infection | 14 days (moderate)  35 days (severe)  Sensitivity analysis:  Uniform(9,19)  Uniform(30,40) | Liu et al [16] |
| LoS of non-COVID patients in ICU |  |  | Lognormal  meanlog = 0.37  sdlog = 0.82  mean = 1.45 days  sd = 2.27 | Fitted distributions to UMCU data from 2014-2017 |
| LoS of non-COVID patients in normal ward |  |  | Weibull  shape = 0.92  scale = 4.18  mean = 4.35 days | Fitted distributions to UMCU data from 2014-2017 |
| LoS of moderately infected patients |  |  | Gamma  shape = 1.88  rate = 0.25  mean = 31.8 days  sd = 30.08 | Fitted distributions to UMCU data from 2020 |
| LoS of severely infected patients |  |  | Gamma  shape = 1.59  rate = 0.05  mean = 7.52 days  sd = 636 | Fitted distributions to UMCU data from 2020 |
| Patient-nurse ratio |  | 1:1 (COVID ICU),  2:1 (COVID ward),  1:1 (Normal ICU),  4:1 (Regular ward) |  | Assumed |
| Patient-doctor ratio |  | 6:1 (COVID ICU, COVID ward, Normal ICU),  10:1 (Regular ward) |  | Assumed |
| Frequency of HCWs visiting patients (ward dependent) |  | Min 10 minutes,  Max 30 minutes |  |  |
| Duty shifts of HCWs per day |  | 3 shifts |  |  |
| Rounds per shift |  | Nurses: 6  Doctors: 2 |  |  |
| Contact rates (per shift) | $c_{n,n}$ | Average number of contacts between nurses | 4·6 | From simulation |
|  | $c_{n,p}$ | Average number of contacts that a nurse has with patients | 19·07 |  |
|  | $c_{n,d}$ | Average number of contacts that a nurse has with doctors | 3 |  |
|  | $c_{p,n}$ | Average number of contacts that a patient has with nurses | $6$ |  |
|  | $c_{p,d}$ | Average number of contacts that a patient has with doctors | 2 |  |
|  | $c_{d,d}$ | Average number of contacts between doctors | 0·43 |  |
|  | $c_{d, p}$ | Average number of contacts a doctor has with patients | 17·4 |  |
|  | $c_{d, n}$ | Average number of contacts a doctor has with nurses | 3 |  |
| Daily arrival rate of non-COVID patients |  | Number of patients that arrive at the hospital per day for non-COVID related reasons | 40 patients per day | Based on UMCU data from 2014-2017 assuming 50% decrease during study period |
| HCW population |  | Constant number of HCWs working in the hospital per day | 870 |  |

* Mean or median values were used from literature; range was used in the sensitivity analyses

**Figure S1. Number of patients admitted to UMCU with a SARS-CoV-2 infection between 27 February and 2 August 2020.** Patients were either admitted to an ICU or to another ward in the hospital.

**Figure S2. Length of stay data of UMCU and fitted distributions for non-COVID and COVID patients in the hospital.** (A)-(B) Histograms show the length of stay distributions for patients admitted to the UMCU between 2014 and 2017. The bold lines represent the fitted probability distributions. The length of stay of patients admitted for non-COVID reasons to the ICUs and to normal wards follow a lognormal distribution and Weibull distribution, respectively. (C)-(D) Histograms show the length of stay distributions for patients admitted with a SARS-CoV-2 infection to the UMCU between 27 February and 24 August 2020. The bold lines represent the fitted probability distributions. The length of stay of patients admitted with a SARS-CoV-2 infection to ICUs and to normal wards follow gamma distributions. The parameters of the probability distributions can be found in Table S1.

**Figure S3. PCR test sensitivity over time since infection**. The empirical estimate based on published data as reported by Grassly et al is shown (black dots) [14]. Two sensitivity analyses were performed: 1) assuming the test sensitivity remains at the maximum after reaching its peak (dark grey dashed) and 2) test sensitivity curve of the main analysis scaled down to 85% (light grey dotted).

**Figure S4. Overview of processes in the agent-based model.** The dashed boxes indicate processes that are only performed for the respective intervention scenario. The time indicates when the process is called (e.g., every 10 min). The smallest time step in the model is 10 minutes.

**Figure S5. Flowchart for patient arrival and patient discharge in the agent-based model.** The time indicates that the process is called every 10 minutes. Note that the smallest time step in the model is 10 minutes.

**Figure S6. Flowchart for HCW community transmission in the agent-based model.** The time indicates that this process is called once a day. Note that the smallest time step in the model is 10 minutes.

**Figure S7. Flowchart for HCW ward change in the agent-based model.** The time indicates that this process is called once a day. Note that the smallest time step in the model is 10 minutes.

****Figure S8. Flowchart for HCWs meeting in common room in the agent-based model.** The time indicates that this process is called every hour. Note that the smallest time step in the model is 10 minutes.

**Figure S9. Flowchart for transition of disease states in agent-based model.** The time indicates that this process is called every 10 minutes. Note that the smallest time step in the model is 10 minutes.

**Figure S10. Flowchart for HCWs visiting patients in the agent-based model.** The time indicates that this process is called every 10 minutes. Note that the smallest time step in the model is 10 minutes.

**Figure S11. Flowchart for contact tracing in the agent-based model.**

**Figure S12. Flowchart for HCW screening in the agent-based model.**
