# Supplementary material for: Interventions to control nosocomial transmission of SARS-CoV-2: a modelling study
Source: BMC Med. 2021 Aug 27;19:211. doi: 10.1186/s12916-021-02060-y (PMC8390112; doi:10.1186/s12916-021-02060-y)
Supplement: Supplementary file 2 — Additional file 2: Supplementary results. (1) Additional results for the main analysis: Table S1. Outcome measures for baseline and intervention scenarios. Figure S1. Positivity rate of screening interventions for different prevalence ranges. Figure S2. Positivity rate of contact tracing interventions for different prevalence ranges. Figure S3. Proportion of detected nosocomial transmissions of the SARS-CoV-2 variant for each simulation scenario. Figure S4. Transmission route contributions for nosocomial transmissions of the SARS-CoV-2 variant for each simulation scenario. Figure S5. Proportion of transmissions from HCWs and from patients for each simulation scenarios. Figure S6. Proportion of nosocomial transmissions in COVID- and non-COVID wards for each simulation scenario. Figure S7. Proportion of transmissions during different infection states for each simulation scenario. (2) Results of sensitivity analyses: Figure S8. Effective reproduction numbers for the nosocomial spread of the SARS-CoV-2 variant for each simulation scenario assuming 50% effective PPE. Figure S9. Number of nosocomial transmissions of the SARS-CoV-2 variant for each simulation scenario assuming 50% effective PPE. Figure S10. Daily percentage of absent HCWs during the hospital epidemic for each simulation scenario assuming 50% effective PPE. Figure S11. Effective reproduction numbers for the nosocomial spread of the SARS-CoV-2 variant for each simulation scenario assuming 70% effective PPE. Figure S12. Number of nosocomial transmissions of the SARS-CoV-2 variant for each simulation scenario assuming 70% effective PPE. Figure S13. Daily percentage of absent HCWs during the hospital epidemic for each simulation scenario assuming 70% effective PPE. Figure S14. Effective reproduction numbers for the nosocomial spread of the SARS-CoV-2 variant for each simulation scenario assuming equal reproduction numbers for symptomatically and asymptomatically infected individuals R_S=1·95 and R_A=1·95. Figur [file 12916_2021_2060_MOESM2_ESM.docx]

**Additional File 2: Supplementary results for the article *Interventions to control nosocomial transmission of SARS-CoV-2: a modelling study***

Thi Mui Pham^1,*,#^, Hannan Tahir^1,*^, Janneke H.H.M. van de Wijgert^1,2^, Bastiaan R. Van der Roest^1^, Pauline Ellerbroek^3^, Marc J.M. Bonten^1,5^, Martin C.J. Bootsma^1,4^, Mirjam E. Kretzschmar^1^

^1^ Julius Center for Health Sciences and Primary Care, University Medical Center Utrecht, Utrecht University, Utrecht, The Netherlands

^2^ Institute of Infection, Veterinary, and Ecological Sciences, University of Liverpool, Liverpool, UK

^3^ Department of Internal Medicine, University Medical Center Utrecht, Utrecht University, Utrecht, The Netherlands

^4^ Department of Mathematics, Faculty of Science, Utrecht University, Utrecht, The Netherlands

^5^ Department of Medical Microbiology, University Medical Center Utrecht, Utrecht University, Utrecht, The Netherlands

**^*^ These authors contributed equally to this work.**

**^#^ Corresponding author:**

Thi Mui Pham

Address: Julius Center for Health Sciences and Primary Care

University Medical Center Utrecht

P.O. Box 85500 Utrecht

The Netherlands

E-mail: [t.m.pham-2@umcutrecht.nl](mailto:t.m.pham-2@umcutrecht.nl)

Telephone: +31648938724

1. **Results on transmission routes**

Our results show that for the considered simulation scenarios most of the nosocomial transmissions of the SARS-CoV-2 variant is mainly driven by transmissions between patients and HCWs (Figure S4). This is expected as we assumed that there is no direct contact between patients and the majority of contacts of HCWs are with patients. Furthermore, for most of the intervention scenarios, over 90% of transmissions occur in non-COVID wards where no use of PPE is assumed in the baseline scenario (Figure S6). Since in our model infected patients are transferred to COVID wards and infected HCWs are assumed to self-isolate immediately upon symptom onset, most transmissions take place during the pre-symptomatic stage of an infected individual (dark-grey bars in Figure S7). This is in line with a French study where secondary cases were exposed mainly in the pre-symptomatic phase [39]. When PPE is used throughout the hospital or HCWs are screened assuming a perfect test sensitivity, most transmissions are prevented (Figure 5 of the main text). In particular, transmissions that occur during non-symptomatic states in non-COVID wards are significantly reduced, decreasing their contribution to the overall number of transmissions (Figure S6-Figure S7).



1. **Results of sensitivity analyses**

We evaluate the changes of our results with respect to changes in our model parameters. We present the results and corresponding plots for the effective reproduction number $R_{E}$, the total number of nosocomial transmissions, and daily number of absent HCWs. The remaining plots can be found online: <https://github.com/htahir2/covid_intra-hospital_model.git>

*PPE effectiveness*

We performed two sensitivity analyses to test the impact of PPE effectiveness values on our results:

1. 50% effective PPE
2. 70% effective PPE

Our sensitivity analyses show that the effective reproduction numbers and the total number of nosocomial transmissions increase with lower PPE effectiveness and decrease with higher PPE effectiveness, in particular for the “PPE in all wards” intervention scenario (compare Figure S8-S7, Figures S11-S12, and Figures 4-5 of the main text). A similar effect can be observed for the daily percentage of HCW absenteeism (compare Figure S10, Figure S13, and Figure 6 of the main text). The relative impact of the different interventions on the reproduction number in comparison to the baseline scenario are similar to what we have observed in our main analysis. The only difference is that for a low value of PPE effectives of 50%, screening every three days with time-invariant perfect sensitivity is more effective in reducing the effective reproduction number, especially for pre-symptomatic HCWs. However, the use of 50% effective PPE in all wards still decreases the effective reproduction number more than the remaining interventions.

*Reproduction number*

We performed a sensitivity analysis to test the impact of equal reproduction numbers of symptomatically and asymptomatically infected individuals on our results (Figure S14-S16). Furthermore, we show the model results for the reproduction numbers resulting from calibrating our model to data on the number of occupied beds by COVID-19 patients at the UMCU (Figure S17-S19). Our sensitivity analyses show that the effective reproduction numbers, the total number of nosocomial transmissions as well as the daily percentage of HCW absenteeism increase with increasing basic reproduction number. In particular, when the reproduction number of asymptomatically infected individuals is as high as the one of symptomatically infected individuals, the respective effective reproduction numbers for asymptomatic patients and HCWs increase. The impact on the overall effective reproduction number is smaller, however, still notable. Qualitatively, our conclusions regarding the relative effect of the considered infection control interventions remain unchanged. For low reproduction numbers as it was the case for the nosocomial spread of the wild-type SARS-CoV-2 variant at UMCU, the numbers of nosocomial transmissions are very small and hence the relative impact of the intervention scenarios in comparison to each other and to the baseline scenario is smaller than for higher reproduction numbers. However, the qualitative conclusions remain unchanged.

*Increased HCW-to-HCW contact rate*

In our main analysis, we assume that HCWs meet other HCWs once every hour. In this sensitivity analysis, we relax this assumption by increasing the contact rates between HCWs to once every 30 minutes and evaluate the impact on our results (Figure S20-S22). The effective reproduction numbers, the total number of nosocomial transmissions, and the daily percentage of HCW absenteeism increase when the contact rate between HCWs is increased. In particular, the effective reproduction numbers for HCWs increase but not those for symptomatic patients (Figure S20). Qualitatively, our conclusions with respect to the impact of the interventions on the hospital epidemic do not change with respect to this parameter.

*Test sensitivity*

We performed two sensitivity analyses:

1. assuming the test sensitivity to remain at the maximum after reaching its peak (high test sensitivity scenario) and
2. reducing the test sensitivity curve of the main analysis by 15% (low test sensitivity scenario).

The respective test sensitivity curves varying from time since infection are shown in Additional File 1: Figure S1. There are only minor differences in our results for both sensitivity scenarios (Figure S23-S25 vs Figures 4-6 of the main text).

*Recovery time*

To test the impact of the recovery time of infected individuals (i.e., the time after which infected individuals are set to non-infectious and recovered in the model), we performed the simulations with a stochastic (instead of fixed) implementation of the recovery times. For this sensitivity analysis we assumed the following uniform distributions for the recovery times:

- Unif(9,19) for asymptomatic and moderately symptomatically infected individuals
- Unif(30,40) for severely symptomatically infected individuals.

The parameters in brackets represent the time since infection and serve as lower and upper bounds in the uniform distribution. Qualitatively, our results do not change with respect to this parameter (Figure S29-S31).

**Table S1. Outcome measures for baseline and intervention scenarios.**

| Scenario | Effective reproduction number* | | | | | Total number of nosocomial transmissions* | Peak percentage of HCW absenteeism (%)^†^ | Peak positivity rates (%)* |
| --- | --- | --- | --- | --- | --- | --- | --- | --- |
|  | Overall | Symptomatic patient | Asymptomatic patient | Pre-symptomatic HCW | Asymptomatic HCW |  |  |  |
| Baseline | 0.65 (0.57, 0.71) | 0.55 (0.46, 0.66) | 0.40 (0.23, 0.60) | 0.94 (0.79, 1.07) | 0.44 (0.33, 0.54) | 526.2 (362.3, 675.2) | 5.4 |  |
| HCW cohorting | 0.62 (0.56, 0.67) | 0.50 (0.40, 0.60) | 0.39 (0.17, 0.60) | 0.91 (0.79, 1.04) | 0.44 (0.31, 0.55) | 457.9 (359, 565.1) | 5.2 |  |
| PPE in all wards | 0.10 (0.07, 0.14) | 0.03 (0.01, 0.06) | 0.03 (0.00, 0.42) | 0.21 (0.13, 0.32) | 0.09 (0.02, 0.18) | 32.9 (21.5, 48.5) | 2.3 |  |
| Screening 3 days perfect sens | 0.24 (0.14, 0.32) | 0.23 (0.10, 0.36) | 0.53 (0.00, 1.23) | 0.28 (0.19, 0.40) | 0.12 (0.03, 0.24) | 90.9 (47, 136.5) | 5.1 | 1.7 (1.6, 1.8) |
| Screening 3 days | 0.59 (0.51, 0.65) | 0.53 (0.42, 0.62) | 0.47 (0.24, 0.71) | 0.81 (0.68, 0.92) | 0.36 (0.22, 0.51) | 419 (298.1, 528.7) | 8.6 | 2.5 (2.4, 2.6) |
| Screening 7 days | 0.63 (0.54, 0.69) | 0.55 (0.47, 0.64) | 0.44 (0.25, 0.64) | 0.87 (0.73, 0.98) | 0.39 (0.29, 0.51) | 473 (353.9, 614.3) | 6.6 | 5.1 (5, 5.3) |
| 7-day Contact tracing perfect sens | 0.44 (0.36, 0.55) | 0.29 (0.21, 0.38) | 0.31 (0.11, 0.61) | 0.74 (0.60, 0.90) | 0.36 (0.22, 0.50) | 232.7 (155.9, 341.4) | 4 | 15.1 (14.1, 16.1) |
| 2-day Contact tracing | 0.41 (0.33, 0.48) | 0.27 (0.18, 0.35) | 0.30 (0.08, 0.65) | 0.71 (0.55, 0.84) | 0.32 (0.19, 0.47) | 202.9 (138.5, 269.6) | 3.6 | 11.3 (9.4, 13.1) |
| 7-day Contact tracing | 0.39 (0.33, 0.44) | 0.25 (0.17, 0.34) | 0.29 (0.05, 0.61) | 0.67 (0.52, 0.80) | 0.31 (0.17, 0.44) | 188.7 (139, 248.6) | 3.9 | 10.4 (9.1, 11.6) |

*Mean values over 100 simulation runs are given. Values in brackets are the lower and upper bounds of 95% uncertainty intervals.

†7-day moving average of mean percentage (over 100 simulation runs)

**Figure S1. Positivity rate of screening interventions for different prevalence ranges.** Results shown are based on $R_{S}$=1.95 and $R_{A}$=0.8 (reproduction numbers for the SARS-CoV-2 variant with 56% higher transmissibility with respect to the wild-type SARS-CoV-2 variant). **(**A) Screening every three days with constant perfect test sensitivity. (B) Screening every three days with imperfect, time-varying test sensitivity. (C) Screening every seven days with imperfect, time-varying test sensitivity. On each day when HCWs were screened, the number of positive tested HCWs among the total number of screened HCWs is computed. The prevalence values on the day when HCWs were screened is divided into categories. For each prevalence category, the positivity rate was computed by the total number of positive tested HCWs divided by the total number of screened HCWs (merging values of all simulations) and is shown as a point. The error bars represent the 95% binomial proportion confidence intervals.

**Figure S2. Positivity rate of contact tracing interventions for different prevalence ranges.** Results shown are based on $R_{S}$=1.95 and $R_{A}$=0.8 (reproduction numbers for the SARS-CoV-2 variant with 56% higher transmissibility with respect to the wild-type SARS-CoV-2 variant). **(**A) Contact tracing of contacts two days prior to symptom onset with perfect test sensitivity. (B) Contact tracing of contacts two days prior to symptom onset with time-varying imperfect test sensitivity. (C) Contact tracing of contacts seven days prior to symptom onset with time-varying, imperfect test sensitivity. For each index case (symptomatically infected HCW), the number of positive contacts and total number of contacts that are traced is computed in each simulation. The prevalence values on the day when an index case was traced, is divided into categories. For each prevalence category, the positivity rate is computed by the total number of positive divided by the total number of traced contacts (all simulations merged) and is shown as a point. The error bars represent the binomial proportion confidence interval.

**Figure S3. Proportion of detected nosocomial transmissions of the SARS-CoV-2 variant for each simulation scenario.** Results shown are based on $R_{S}$=1.95 and $R_{A}$=0.8 (reproduction numbers for the SARS-CoV-2 variant with 56% higher transmissibility with respect to the wild-type SARS-CoV-2 variant). The colored rectangular bars with black borders represent the mean proportion of patients detected with a SARS-CoV-2 infection in the hospital due to symptom onset or detection by an intervention (over 100 simulation runs). The denominator are patients either admitted with a SARS-CoV-2 infection (asymptomatic or pre-symptomatic) or acquired it in the hospital. The proportions of infected patients undetected comprise patients who are discharged to community in a pre-symptomatic or asymptomatic state. The grey error bars the respective 95% uncertainty intervals.

**Figure S4. Transmission route contributions for nosocomial transmissions of the SARS-CoV-2 variant for each simulation scenario**. Three different transmission routes are considered: From patient to HCW (Patient-HCW), from HCW to patient (HCW-patient), and from HCW to HCW (HCW-HCW). The colored rectangular bars represent the mean percentage of transmissions (averaged over 100 simulations) due to the respective transmission route for each simulation scenario. The grey error bars represent the respective 95% uncertainty intervals. For screening every 3 days and 7-day contact tracing, we considered two different test sensitivity scenarios: time-invariant perfect test sensitivity (perfect sens) and time-varying imperfect test sensitivity.

**Figure S5. Proportion of transmissions from HCWs and from patients for each simulation scenarios.** Mean percentage of total transmissions (averaged over 100 simulation runs) that occurred from HCWs vs from patients are shown in stacked bar plots. Results shown are based on $R_{S}$=1.95 and $R_{A}$=0.8 (reproduction numbers for the SARS-CoV-2 variant with 56% higher transmissibility with respect to the wild-type SARS-CoV-2 variant).

**Figure S6. Proportion of nosocomial transmissions in COVID- and non-COVID wards for each simulation scenario.** The mean percentages of nosocomial transmissions (averaged over 100 simulation runs) in COVID and non-COVID wards are shown in stacked bar plots. Results shown are based on $R_{S}$=1.95 and $R_{A}$=0.8 (reproduction numbers for the SARS-CoV-2 variant with 56% higher transmissibility with respect to the wild-type SARS-CoV-2 variant).

**Figure S7. Proportion of transmissions during different infection states for each simulation scenario.** The mean percentages of transmissions (averaged over 100 simulations) that occurred while the infected individual was in an asymptomatic, pre-symptomatic, or symptomatic state are shown in stacked bar plots. Results shown are based on $R_{S}$=*1.95* and $R_{A}$=*0.8* (reproduction numbers for the SARS-CoV-2 variant with 56% higher transmissibility with respect to the wild-type SARS-CoV-2 variant).

**Figure S8. Effective reproduction numbers for the nosocomial spread of the SARS-CoV-2 variant for each simulation scenario assuming 50% effective PPE.** Results shown are based on $R_{S}$=1.95 and $R_{A}$=0.8 (reproduction numbers for the SARS-CoV-2 variant with 56% higher transmissibility with respect to the wild-type SARS-CoV-2 variant). (A) For each simulation scenario, violin and boxplots of the overall reproduction numbers (for pre-/symptomatic and asymptomatic patients and HCWs combined) are shown (over 100 simulations). (B) For each simulation scenario, violin and boxplots of the reproduction numbers for pre-/symptomatic and asymptomatic individuals are shown (over 100 simulations). Note that since HCWs are assumed to immediately self-isolate upon symptom onset, the reproduction number is assigned to the pre-symptomatic state. The horizontal dashed line represents a reproduction number of 1. For screening every three days and contact tracing seven days prior to symptom onset of SARS-CoV-2 infected HCWs, we considered two different test sensitivity scenarios: time-invariant perfect test sensitivity (perfect sens) and imperfect test sensitivity varying from time since infection.

**Figure S9. Number of nosocomial transmissions of the SARS-CoV-2 variant for each simulation scenario assuming 50% effective PPE.** Results shown are based on $R_{S}$=*1.95* and $R_{A}$=*0.8* (reproduction numbers for the SARS-CoV-2 variant with 56% higher transmissibility with respect to the wild-type SARS-CoV-2 variant). The full rectangular bar height represents the mean total number of nosocomial transmissions during the whole study period (over 100 simulation runs). The grey error bars represent the corresponding 95% uncertainty intervals. Patients that acquire a SARS-CoV-2 nosocomial infection may be diagnosed in the hospital (due to symptom onset during hospital stay or due to detection by an intervention) or discharged to the community in a pre-symptomatic or asymptomatic state. The rectangular bars with the black border represent the mean number of individuals (patients and HCWs) infected with SARS-CoV-2 and diagnosed in the hospital. The lighter rectangular bars represent the remaining mean number of patients discharged to community undiagnosed. For screening every 3 days and 7-day contact tracing, we considered two different test sensitivity scenarios: time-invariant perfect test sensitivity (perfect sens) and time-varying imperfect test sensitivity.

**Figure S10. Daily percentage of absent HCWs during the hospital epidemic for each simulation scenario assuming 50% effective PPE.** Results shown are based on R_S=1.95 and R_A=0.8 (reproduction numbers for the SARS-CoV-2 variant with 56% higher transmissibility with respect to the wild-type SARS-CoV-2 variant). The 7-day moving average of the mean percentage (over 100 simulation runs) of HCWs absent from work due to symptom onset or a detected SARS-CoV-2 infection screening or contact tracing is shown. For screening every 3 days and contact tracing 7 days prior to symptom onset of SARS-CoV-2 infected HCWs, we considered two different test sensitivity scenarios: time-invariant perfect test sensitivity (perfect sens) and time-varying imperfect test sensitivity.

**Figure S11. Effective reproduction numbers for the nosocomial spread of the SARS-CoV-2 variant for each simulation scenario assuming 70% effective PPE.** Results shown are based on $R_{S}$=1.95 and $R_{A}$=0.8 (reproduction numbers for the SARS-CoV-2 variant with 56% higher transmissibility with respect to the wild-type SARS-CoV-2 variant). (A) For each simulation scenario, violin and boxplots of the overall reproduction numbers (for pre-/symptomatic and asymptomatic patients and HCWs combined) are shown (over 100 simulations). (B) For each simulation scenario, violin and boxplots of the reproduction numbers for pre-/symptomatic and asymptomatic individuals are shown (over 100 simulations). Note that since HCWs are assumed to immediately self-isolate upon symptom onset, the reproduction number is assigned to the pre-symptomatic state. The horizontal dashed line represents a reproduction number of 1. For screening every three days and contact tracing seven days prior to symptom onset of SARS-CoV-2 infected HCWs, we considered two different test sensitivity scenarios: time-invariant perfect test sensitivity (perfect sens) and imperfect test sensitivity varying from time since infection.

**Figure S12. Number of nosocomial transmissions of the SARS-CoV-2 variant for each simulation scenario assuming 70% effective PPE.** Results shown are based on $R_{S}$=*1.95* and $R_{A}$=*0.8* (reproduction numbers for the SARS-CoV-2 variant with 56% higher transmissibility with respect to the wild-type SARS-CoV-2 variant). The full rectangular bar height represents the mean total number of nosocomial transmissions during the whole study period (over 100 simulation runs). The grey error bars represent the corresponding 95% uncertainty intervals. Patients that acquire a SARS-CoV-2 nosocomial infection may be diagnosed in the hospital (due to symptom onset during hospital stay or due to detection by an intervention) or discharged to the community in a pre-symptomatic or asymptomatic state. The rectangular bars with the black border represent the mean number of individuals (patients and HCWs) infected with SARS-CoV-2 and diagnosed in the hospital. The lighter rectangular bars represent the remaining mean number of patients discharged to community undiagnosed. For screening every 3 days and 7-day contact tracing, we considered two different test sensitivity scenarios: time-invariant perfect test sensitivity (perfect sens) and time-varying imperfect test sensitivity.

**

**Figure S13. Daily percentage of absent HCWs during the hospital epidemic for each simulation scenario assuming 70% effective PPE.** Results shown are based on R_S=1.95 and R_A=0.8 (reproduction numbers for the SARS-CoV-2 variant with 56% higher transmissibility with respect to the wild-type SARS-CoV-2 variant). The 7-day moving average of the mean percentage (over 100 simulation runs) of HCWs absent from work due to symptom onset or a detected SARS-CoV-2 infection screening or contact tracing is shown. For screening every 3 days and contact tracing 7 days prior to symptom onset of SARS-CoV-2 infected HCWs, we considered two different test sensitivity scenarios: time-invariant perfect test sensitivity (perfect sens) and time-varying imperfect test sensitivity.

**Figure S14. Effective reproduction numbers for the nosocomial spread of the SARS-CoV-2 variant for each simulation scenario assuming equal reproduction numbers for symptomatically and asymptomatically infected individuals** $\boldsymbol{R}_{\boldsymbol{S}}$**=1.95 and** $\boldsymbol{R}_{\boldsymbol{A}}$**=1.95.** (A) For each simulation scenario, violin and boxplots of the overall reproduction numbers (for pre-/symptomatic and asymptomatic patients and HCWs combined) are shown (over 100 simulations). (B) For each simulation scenario, violin and boxplots of the reproduction numbers for pre-/symptomatic and asymptomatic individuals are shown (over 100 simulations). Note that since HCWs are assumed to immediately self-isolate upon symptom onset, the reproduction number is assigned to the pre-symptomatic state. The horizontal dashed line represents a reproduction number of 1. For screening every three days and contact tracing seven days prior to symptom onset of SARS-CoV-2 infected HCWs, we considered two different test sensitivity scenarios: time-invariant perfect test sensitivity (perfect sens) and imperfect test sensitivity varying from time since infection.

**Figure S15. Number of nosocomial transmissions of the SARS-CoV-2 variant for each simulation scenario assuming equal reproduction numbers for symptomatically and asymptomatically infected individuals** $\boldsymbol{R}_{\boldsymbol{S}}$**=1.95 and** $\boldsymbol{R}_{\boldsymbol{A}}$**=1.95.** The full rectangular bar height represents the mean total number of nosocomial transmissions during the whole study period (over 100 simulation runs). The grey error bars represent the corresponding 95% uncertainty intervals. Patients that acquire a SARS-CoV-2 nosocomial infection may be diagnosed in the hospital (due to symptom onset during hospital stay or due to detection by an intervention) or discharged to the community in a pre-symptomatic or asymptomatic state. The rectangular bars with the black border represent the mean number of individuals (patients and HCWs) infected with SARS-CoV-2 and diagnosed in the hospital. The lighter rectangular bars represent the remaining mean number of patients discharged to community undiagnosed. For screening every 3 days and 7-day contact tracing, we considered two different test sensitivity scenarios: time-invariant perfect test sensitivity (perfect sens) and time-varying imperfect test sensitivity.

**

**Figure S16. Daily percentage of absent HCWs during the hospital epidemic for each simulation scenario assuming equal reproduction numbers for symptomatic and asymptomatic individuals** $\boldsymbol{R}_{\boldsymbol{S}}$**=1.95 and** $\boldsymbol{R}_{\boldsymbol{A}}$**=1.95.** The 7-day moving average of the mean percentage (over 100 simulation runs) of HCWs absent from work due to symptom onset or a detected SARS-CoV-2 infection screening or contact tracing is shown. For screening every 3 days and contact tracing 7 days prior to symptom onset of SARS-CoV-2 infected HCWs, we considered two different test sensitivity scenarios: time-invariant perfect test sensitivity (perfect sens) and imperfect test sensitivity from time since infection.

**Figure S17. Effective reproduction numbers for each simulation scenario assuming reproduction numbers of the wild-type SARS-CoV-2 variant.** Results shown are based on $R_{S}$=1.25 and $R_{A}$=0.5. (A) For each simulation scenario, violin and boxplots of the overall reproduction numbers (for pre-/symptomatic and asymptomatic patients and HCWs combined) are shown (over 100 simulations). (B) For each simulation scenario, violin and boxplots of the reproduction numbers for pre-/symptomatic and asymptomatic individuals are shown (over 100 simulations). Note that since HCWs are assumed to immediately self-isolate upon symptom onset, the reproduction number is assigned to the pre-symptomatic state. The horizontal dashed line represents a reproduction number of 1. For screening every three days and contact tracing seven days prior to symptom onset of SARS-CoV-2 infected HCWs, we considered two different test sensitivity scenarios: time-invariant perfect test sensitivity (perfect sens) and imperfect test sensitivity varying from time since infection.

**Figure S18. Number of nosocomial transmissions of the SARS-CoV-2 variant for each simulation scenario assuming reproduction numbers of the wild-type SARS-CoV-2 variant.** Results shown are based on $R_{S}$=1.25 and $R_{A}$=0.5. The full rectangular bar height represents the mean total number of nosocomial transmissions during the whole study period (over 100 simulation runs). The grey error bars represent the corresponding 95% uncertainty intervals. Patients that acquire a SARS-CoV-2 nosocomial infection may be diagnosed in the hospital (due to symptom onset during hospital stay or due to detection by an intervention) or discharged to the community in a pre-symptomatic or asymptomatic state. The rectangular bars with the black border represent the mean number of individuals (patients and HCWs) infected with SARS-CoV-2 and diagnosed in the hospital. The lighter rectangular bars represent the remaining mean number of patients discharged to community undiagnosed. For screening every 3 days and 7-day contact tracing, we considered two different test sensitivity scenarios: time-invariant perfect test sensitivity (perfect sens) and time-varying imperfect test sensitivity.

**Figure S19. Daily percentage of absent HCWs during the hospital epidemic for each simulation scenario assuming reproduction numbers of the wild-type SARS-CoV-2 variant.** Results shown are based on $R_{S}$=1.25 and $R_{A}$=0.5. The 7-day moving average of the mean percentage (over 100 simulation runs) of HCWs absent from work due to symptom onset or a detected SARS-CoV-2 infection screening or contact tracing is shown. For screening every 3 days and contact tracing 7 days prior to symptom onset of SARS-CoV-2 infected HCWs, we considered two different test sensitivity scenarios: time-invariant perfect test sensitivity (perfect sens) and imperfect test sensitivity from time since infection.

**Figure S20. Effective reproduction numbers for the nosocomial spread of the SARS-CoV-2 variant for each simulation scenario assuming higher contact rates between HCWs.** Results shown are based on $R_{S}$=1.95 and $R_{A}$=0.8 (reproduction numbers for the SARS-CoV-2 variant with 56% higher transmissibility with respect to the wild-type SARS-CoV-2 variant). (A) For each simulation scenario, violin and boxplots of the overall reproduction numbers (for pre-/symptomatic and asymptomatic patients and HCWs combined) are shown (over 100 simulations). (B) For each simulation scenario, violin and boxplots of the reproduction numbers for pre-/symptomatic and asymptomatic individuals are shown (over 100 simulations). Note that since HCWs are assumed to immediately self-isolate upon symptom onset, the reproduction number is assigned to the pre-symptomatic state. The horizontal dashed line represents a reproduction number of 1. For screening every three days and contact tracing seven days prior to symptom onset of SARS-CoV-2 infected HCWs, we considered two different test sensitivity scenarios: time-invariant perfect test sensitivity (perfect sens) and imperfect test sensitivity varying from time since infection.

**Figure S21. Number of nosocomial transmissions of the SARS-CoV-2 variant for each simulation scenario assuming higher contact rates between HCWs.** Results shown are based on $R_{S}$=1.95 and $R_{A}$=0.8 (reproduction numbers for the SARS-CoV-2 variant with 56% higher transmissibility with respect to the wild-type SARS-CoV-2 variant). The full rectangular bar height represents the mean total number of nosocomial transmissions during the whole study period (over 100 simulation runs). The grey error bars represent the corresponding 95% uncertainty intervals. Patients that acquire a SARS-CoV-2 nosocomial infection may be diagnosed in the hospital (due to symptom onset during hospital stay or due to detection by an intervention) or discharged to the community in a pre-symptomatic or asymptomatic state. The rectangular bars with the black border represent the mean number of individuals (patients and HCWs) infected with SARS-CoV-2 and diagnosed in the hospital. The lighter rectangular bars represent the remaining mean number of patients discharged to community undiagnosed. For screening every 3 days and 7-day contact tracing, we considered two different test sensitivity scenarios: time-invariant perfect test sensitivity (perfect sens) and time-varying imperfect test sensitivity.

**Figure S22. Daily percentage of absent HCWs during the hospital epidemic for each simulation scenario assuming higher contact rates between HCWs.** Results shown are based on $R_{S}$=1.95 and $R_{A}$=0.8 (reproduction numbers for the SARS-CoV-2 variant with 56% higher transmissibility with respect to the wild-type SARS-CoV-2 variant). The 7-day moving average of the mean percentage (over 100 simulation runs) of HCWs absent from work due to symptom onset or a detected SARS-CoV-2 infection screening or contact tracing is shown. For screening every 3 days and contact tracing 7 days prior to symptom onset of SARS-CoV-2 infected HCWs, we considered two different test sensitivity scenarios: time-invariant perfect test sensitivity (perfect sens) and imperfect test sensitivity from time since infection.

**Figure S23. Effective reproduction numbers for the nosocomial spread of the SARS-CoV-2 variant for the high test sensitivity scenario.** Results shown are based on $R_{S}$=1.95 and $R_{A}$=0.8 (reproduction numbers for the SARS-CoV-2 variant with 56% higher transmissibility with respect to the wild-type SARS-CoV-2 variant). (A) For each simulation scenario, violin and boxplots of the overall reproduction numbers (for pre-/symptomatic and asymptomatic patients and HCWs combined) are shown (over 100 simulations). (B) For each simulation scenario, violin and boxplots of the reproduction numbers for pre-/symptomatic and asymptomatic individuals are shown (over 100 simulations). Note that since HCWs are assumed to immediately self-isolate upon symptom onset, the reproduction number is assigned to the pre-symptomatic state. The horizontal dashed line represents a reproduction number of 1. For screening every three days and contact tracing seven days prior to symptom onset of SARS-CoV-2 infected HCWs, we considered two different test sensitivity scenarios: time-invariant perfect test sensitivity (perfect sens) and imperfect test sensitivity varying from time since infection.

**Figure S24. Number of nosocomial transmissions of the SARS-CoV-2 variant for each simulation scenario for the high test sensitivity scenario.** Results shown are based on $R_{S}$=1.95 and $R_{A}$=0.8 (reproduction numbers for the SARS-CoV-2 variant with 56% higher transmissibility with respect to the wild-type SARS-CoV-2 variant). The full rectangular bar height represents the mean total number of nosocomial transmissions during the whole study period (over 100 simulation runs). The grey error bars represent the corresponding 95% uncertainty intervals. Patients that acquire a SARS-CoV-2 nosocomial infection may be diagnosed in the hospital (due to symptom onset during hospital stay or due to detection by an intervention) or discharged to the community in a pre-symptomatic or asymptomatic state. The rectangular bars with the black border represent the mean number of individuals (patients and HCWs) infected with SARS-CoV-2 and diagnosed in the hospital. The lighter rectangular bars represent the remaining mean number of patients discharged to community undiagnosed. For screening every 3 days and 7-day contact tracing, we considered two different test sensitivity scenarios: time-invariant perfect test sensitivity (perfect sens) and time-varying imperfect test sensitivity.

**Figure S25. Daily percentage of absent HCWs during the hospital epidemic for each simulation scenario for the high test sensitivity scenario.** Results shown are based on $R_{S}$=1.95 and $R_{A}$=0.8 (reproduction numbers for the SARS-CoV-2 variant with 56% higher transmissibility with respect to the wild-type SARS-CoV-2 variant). The 7-day moving average of the mean percentage (over 100 simulation runs) of HCWs absent from work due to symptom onset or a detected SARS-CoV-2 infection screening or contact tracing is shown. For screening every 3 days and contact tracing 7 days prior to symptom onset of SARS-CoV-2 infected HCWs, we considered two different test sensitivity scenarios: time-invariant perfect test sensitivity (perfect sens) and imperfect test sensitivity from time since infection.

**Figure S26. Effective reproduction numbers for the nosocomial spread of the SARS-CoV-2 variant for the low test sensitivity scenario.** Results shown are based on $R_{S}$=1.95 and $R_{A}$=0.8 (reproduction numbers for the SARS-CoV-2 variant with 56% higher transmissibility with respect to the wild-type SARS-CoV-2 variant). (A) For each simulation scenario, violin and boxplots of the overall reproduction numbers (for pre-/symptomatic and asymptomatic patients and HCWs combined) are shown (over 100 simulations). (B) For each simulation scenario, violin and boxplots of the reproduction numbers for pre-/symptomatic and asymptomatic individuals are shown (over 100 simulations). Note that since HCWs are assumed to immediately self-isolate upon symptom onset, the reproduction number is assigned to the pre-symptomatic state. The horizontal dashed line represents a reproduction number of 1. For screening every three days and contact tracing seven days prior to symptom onset of SARS-CoV-2 infected HCWs, we considered two different test sensitivity scenarios: time-invariant perfect test sensitivity (perfect sens) and imperfect test sensitivity varying from time since infection.

**Figure S27. Number of nosocomial transmissions of the SARS-CoV-2 variant for each simulation scenario for the low test sensitivity scenario.** Results shown are based on $R_{S}$=1.95 and $R_{A}$=0.8 (reproduction numbers for the SARS-CoV-2 variant with 56% higher transmissibility with respect to the wild-type SARS-CoV-2 variant). The full rectangular bar height represents the mean total number of nosocomial transmissions during the whole study period (over 100 simulation runs). The grey error bars represent the corresponding 95% uncertainty intervals. Patients that acquire a SARS-CoV-2 nosocomial infection may be diagnosed in the hospital (due to symptom onset during hospital stay or due to detection by an intervention) or discharged to the community in a pre-symptomatic or asymptomatic state. The rectangular bars with the black border represent the mean number of individuals (patients and HCWs) infected with SARS-CoV-2 and diagnosed in the hospital. The lighter rectangular bars represent the remaining mean number of patients discharged to community undiagnosed. For screening every 3 days and 7-day contact tracing, we considered two different test sensitivity scenarios: time-invariant perfect test sensitivity (perfect sens) and time-varying imperfect test sensitivity.

**Figure S28. Daily percentage of absent HCWs during the hospital epidemic for each simulation scenario for the low test sensitivity scenario.** Results shown are based on $R_{S}$=1.95 and $R_{A}$=0.8 (reproduction numbers for the SARS-CoV-2 variant with 56% higher transmissibility with respect to the wild-type SARS-CoV-2 variant). The 7-day moving average of the mean percentage (over 100 simulation runs) of HCWs absent from work due to symptom onset or a detected SARS-CoV-2 infection screening or contact tracing is shown. For screening every 3 days and contact tracing 7 days prior to symptom onset of SARS-CoV-2 infected HCWs, we considered two different test sensitivity scenarios: time-invariant perfect test sensitivity (perfect sens) and imperfect test sensitivity from time since infection.

**Figure S29. Effective reproduction numbers for the nosocomial spread of the SARS-CoV-2 variant for the recovery time sensitivity scenario.** Results shown are based on $R_{S}$=1.95 and $R_{A}$=0.8 (reproduction numbers for the SARS-CoV-2 variant with 56% higher transmissibility with respect to the wild-type SARS-CoV-2 variant). (A) For each simulation scenario, violin and boxplots of the overall reproduction numbers (for pre-/symptomatic and asymptomatic patients and HCWs combined) are shown (over 100 simulations). (B) For each simulation scenario, violin and boxplots of the reproduction numbers for pre-/symptomatic and asymptomatic individuals are shown (over 100 simulations). Note that since HCWs are assumed to immediately self-isolate upon symptom onset, the reproduction number is assigned to the pre-symptomatic state. The horizontal dashed line represents a reproduction number of 1. For screening every three days and contact tracing seven days prior to symptom onset of SARS-CoV-2 infected HCWs, we considered two different test sensitivity scenarios: time-invariant perfect test sensitivity (perfect sens) and imperfect test sensitivity varying from time since infection.

**Figure S30. Number of nosocomial transmissions of the SARS-CoV-2 variant for each simulation scenario for the recovery time sensitivity scenario.** Results shown are based on $R_{S}$=1.95 and $R_{A}$=0.8 (reproduction numbers for the SARS-CoV-2 variant with 56% higher transmissibility with respect to the wild-type SARS-CoV-2 variant). The full rectangular bar height represents the mean total number of nosocomial transmissions during the whole study period (over 100 simulation runs). The grey error bars represent the corresponding 95% uncertainty intervals. Patients that acquire a SARS-CoV-2 nosocomial infection may be diagnosed in the hospital (due to symptom onset during hospital stay or due to detection by an intervention) or discharged to the community in a pre-symptomatic or asymptomatic state. The rectangular bars with the black border represent the mean number of individuals (patients and HCWs) infected with SARS-CoV-2 and diagnosed in the hospital. The lighter rectangular bars represent the remaining mean number of patients discharged to community undiagnosed. For screening every 3 days and 7-day contact tracing, we considered two different test sensitivity scenarios: time-invariant perfect test sensitivity (perfect sens) and time-varying imperfect test sensitivity.

**

**Figure S31. Daily percentage of absent HCWs during the hospital epidemic for each simulation scenario for the recovery time sensitivity scenario.** Results shown are based on $R_{S}$=1.95 and $R_{A}$=0.8 (reproduction numbers for the SARS-CoV-2 variant with 56% higher transmissibility with respect to the wild-type SARS-CoV-2 variant). The 7-day moving average of the mean percentage (over 100 simulation runs) of HCWs absent from work due to symptom onset or a detected SARS-CoV-2 infection screening or contact tracing is shown. For screening every 3 days and contact tracing 7 days prior to symptom onset of SARS-CoV-2 infected HCWs, we considered two different test sensitivity scenarios: time-invariant perfect test sensitivity (perfect sens) and imperfect test sensitivity from time since infection.
